# TCR-T cell therapy for solid tumors: challenges and emerging solutions

**DOI:** 10.3389/fphar.2025.1493346

**Published:** 2025-03-10

**Authors:** Wanjun He, Kai Cui, Muhammad Asad Farooq, Na Huang, Songshan Zhu, Dan Jiang, Xiqian Zhang, Jian Chen, Yinxia Liu, Guangxian Xu

**Affiliations:** ^1^ Guangdong Provincial Key Laboratory of Medical Immunology and Molecular Diagnostics, The First Dongguan Affiliated Hospital, School of Medical Technology, Guangdong Medical University, Dongguan, China; ^2^ Dongguan Key Laboratory of Molecular Immunology and Cell Therapy, Guangdong Medical University, Dongguan, China; ^3^ Yinchuan Guolong Orthopedic Hospital, Yinchuan, China

**Keywords:** adoptive cell therapy, immunotherapy, solid tumor, TCR, TCR-T cell

## Abstract

With the use of T cell receptor T cells (TCR-T cells) and chimeric antigen receptor T cells (CAR-T cells), T-cell immunotherapy for cancer has advanced significantly in recent years. CAR-T cell therapy has demonstrated extraordinary success when used to treat hematologic malignancies. Nevertheless, there are several barriers that prevent this achievement from being applied to solid tumors, such as challenges with tumor targeting and inadequate transit and adaption of genetically modified T-cells, especially in unfavorable tumor microenvironments The deficiencies of CAR-T cell therapy in the treatment of solid tumors are compensated for by TCR-T cells, which have a stronger homing ability to initiate intracellular commands, 90% of the proteins can be used as developmental targets, and they can recognize target antigens more broadly. As a result, TCR-T cells may be more effective in treating solid tumors. In this review, we discussed the structure of TCR-T and have outlined the drawbacks of TCR-T in cancer therapy, and suggested potential remedies. This review is crucial in understanding the current state and future potential of TCR-T cell therapy. We emphasize how important it is to use combinatorial approaches, combining new combinations of various emerging strategies with over-the-counter therapies designed for TCR-T, to increase the anti-tumor efficacy of TCR-T inside the TME and maximize treatment safety, especially when it comes to solid tumor immunotherapies.

## 1 Introduction

Cancer treatment is still a major global concern, and standard approaches, including surgery, radiation, and chemotherapy, are frequently associated with metastasis and recurrence. Therefore, it is essential to develop new anti-cancer therapies. By genetically altering human immune cells to accurately detect and eliminate malignant cells, immune cell therapy has completely changed traditional methods while minimizing collateral damage to healthy organs. As such, it now forms the fourth essential cornerstone of cancer treatment, following radiation, surgery, and targeted therapy. T-cell therapy has made significant strides in recent years (Mo et al., 2017), establishing it as a key treatment strategy for cancer patients.

CAR-T cell therapy, at the forefront of therapeutic innovation, has numerous authorized medications that target different hematological cancers, such as lymphoma and myeloma (Wang et al., 2021; Hu et al., 2021). CAR-T cell therapy in hematological malignancies has brought new life hope to many patients who were previously difficult to treat. However, the application of this therapy in the treatment of solid tumors faces many challenges, including the complex microenvironment of solid tumors, high antigenic heterogeneity, and the difficulty of effective penetration of CAR T cells into the tumor (Zhao and Cao, 2019). Pancreatic carcinoma (PC) is one of the most common malignancies. In an investigation, 4.1R can suppress the anti-tumor activity of T cell responses. And overcome the problem of tumor-specific targets. The absence of 4.1R in natural killer group 2D (NKG2D) -CAR T cells enables to overcome the problem of tumor-specific targets and have stronger proliferation and killing function, providing a potential therapeutic strategy for the clinical treatment of PC (Gao et al., 2021). In one study, a CAR T cell co-expressing CXCR5 and IL-7 (C5/IL7-CAR-T) was designed to enhance the survival of CAR T cells and reduce cell depletion and apoptosis through the pSTAT5 signaling pathway, showing significant efficacy in the treatment of osteosarcoma (Hui et al., 2024). Another study delineates a new type of CAR T cell therapy based on stem cell-like T cells (T_STEM_), which has a greater ability to expand compared to traditional T cell-based CAR T cells. Five days after a single infusion in the best patient of the same batch, an MRI scan showed almost complete regression of the solid tumor (Choi et al., 2024). This is an exciting milestone on the road to cancer treatment, given that blood tumors have been largely conquered by cell therapy and solid tumors have been left in the dark. This research results not only validate the feasibility of CAR-T cell therapy in the treatment of solid tumors. More importantly, it shows us the great potential of this therapy to rapidly and significantly reduce the size of tumors.

With research endeavors in solid tumor treatment progressively surging year by year (Table 1), TCR-T cell therapy is the optimal alternative to the CAR-T cell therapy regimen, amalgamating numerous advantages into one comprehensive approach. Given the ability of major histocompatibility complex (MHC) molecules to present peptide chains derived from both cell surface and intracellular proteins, TCR-T cell therapy enjoys an inherent advantage over CAR-T cell therapy by targeting a broader spectrum of antigens. In 1988, Blüthmann et al. ushered in a new era of TCR gene therapy by introducing a novel TCR gene into conventional T cells, thereby endowing the modified T cells with identical antigenic specificity (Blüthmann et al., 1988). In both hematological and solid tumor treatments, TCR-T cell therapy has exhibited a favorable safety and efficacy profile (Garber, 2018; Chapuis et al., 2019). Thus far, the emphasis of TCR-T cell therapy has primarily centered on solid tumors, and more and more new targets have been discovered (Ma et al., 2024; Ullah et al., 2024a). This includes the recognition of tumor-specific intracellular proteins, thereby enhancing the capacity of the targeted antigen pool to encompass tumor self-proteins (Tran et al., 2017; Vormehr et al., 2016; Schumacher and Schreiber, 2015), neoantigens resulting from tumor-specific random mutations, and cancer testicular antigens. With the added benefit that genital tissues lack MHC molecules, these antigens are typically only expressed in specific tumors and genital tissues. As a result, TCR-T cell therapy has a broader range of applications in solid tumors, which improves treatment effectiveness. Moreover, researchers find it easier to identify tumor antigens with high specificity, rendering the process of TCR-T cell therapy safer and associated with a reduced incidence of therapeutic side effects, such as neurotoxicity. This presents an opportunity to position TCR-T as a viable alternative to CAR-T cell therapy in treating solid tumors (Garber, 2018). The cancer/testis antigen New York esophageal squamous cell carcinoma 1 (NY-ESO-1), renowned for its high immunogenicity and widespread expression, stands out as an exemplary target antigen for TCR-T cell therapy. TCR-T cell therapy specifically targeting NY-ESO-1 can treat multiple myeloma (Rapoport et al., 2015), metastatic melanoma, metastatic synovial sarcoma (Robbins et al., 2011; Robbins et al., 2015; D'Angelo et al., 2018), provided antigen-specific and multifunctional activity, durable antitumor responses, and showed promising results. Nevertheless, inadequate *in vivo* persistence of infused T cells during early clinical trials resulted in the inability to demonstrate clinical efficacy in certain patients with solid tumors (Michalek et al., 2007; Fraietta et al., 2018). For patients experiencing advanced relapse, TCR-T cell therapy exhibits diminished efficacy in infiltrating due to the heightened immunosuppressive nature of the TME (Hafezi et al., 2021). While TCR-T cells targeting tumor antigens hold promise for treating solid tumors, numerous potential obstacles persist, underscoring the imperative for ongoing enhancements in TCR-T cell therapy.

**TABLE 1:** Current clinical targets of TCR-T cell therapy for solid tumors (ClinicalTrials.gov).

Antigen target	Type of antigen	Clinical trial number	Start	Complete	Phase	Country	HLA^a^	Status	Conditions
HPV^b^	TSA^j^	NCT05357027 NCT05686226 NCT05639972 NCT04015336 NCT04411134 NCT05973487	2022.08 2023.03 2024.06 2020.07 2020.05 2024.02	2024.08 2025.01 2026.10 2020.07 2020.07 2026.12	ⅠⅡ Ⅱ ⅠⅡ Ⅱ Ⅰ Ⅰ	China United States United States United States United States United States	HLA-A2 HLA-A*02:01 HLA-A*02:01 HLA-A*02:01 HLA-A*02:01 HLA-C*07:02, HLA-A*02:01 and HLA-C*07:02 plus HLA-A*02:01	Recruiting Recruiting Not yet recruiting Terminated Withdrawn Recruiting	Cervical Carcinoma Cervical Cancer Throat Cancer Oropharynx Cancer
HBV^c^	TSA	NCT05905731 NCT04745403	2023.06 2022.05	2026.06 2028.07	Ⅰ Ⅰ	China Singapore	— HLA-A*02:01/24:02	Active, not recruiting Recruiting	Chronic Hepatitis B Hepatocellular Carcinoma
EBV^d^	TSA	NCT06135922 NCT04156217 NCT06119256 NCT05587543 NCT04509726	2023.08 2020.02 2023.08 2022.12 2023.03	2026.12 2021.10 2026.12 2030.10 2023.08	Ⅰ Ⅰ Ⅰ Ⅰ ⅠⅡ	China China China China China		Recruiting Completed Recruiting Recruiting Recruiting	EBV-associated Hemophagocytic Lymphohistiocytosis EBV Infection EBV Emia and EBV Positive PTLD After Allogenic HSCT
CMV^e^	TSA	NCT05140187 NCT05089838	2021.10 2021.01	2024.12 2023.10	Ⅰ Ⅰ	China China	HLA-A*11:01/02:01/24:02	Recruiting Unknown status	CMV Infection After Allogenic HSCT Allogeneic Hematopoietic Stem Cell Transplantation CMV Infection
KRAS^f^	TSA	NCT05438667 NCT04146298 NCT05933668 NCT06043713 NCT06218914 NCT06253520 NCT06105021	2022.06 2021.10 2023.07 2023.12 2024.02 2024.03 2024.02	2026.05 2025.03 2026.07 2025.12 2040.01 2033.06 2029.12	Ⅰ ⅠⅡ Ⅰ Ⅰ Ⅰ Ⅰ ⅠⅡ	China China China United States United States United States United States	HLA-A*11:01 HLA-A*11:01 HLA-A*11:01 HLA-A*11:01 HLA-C*08:02 — HLA-A*11:01	Recruiting Recruiting Not yet recruiting Recruiting Recruiting Recruiting Recruiting	Pancreatic Cancer Pancreatic Neoplasms Pancreatic Ductal Adenocarcinoma
MAGE^g^	CGA^k^	NCT04729543	2020.10	2027.10	ⅠⅡ	Netherlands	HLA-A2*0,201	Recruiting	Melanoma Melanoma, Uveal Head and Neck Cancer
NY-ESO-1	CGA	NCT05881525 NCT05989828	2023.06 2024.04	2025.03 2027.04	Ⅰ Ⅰ	China United States	HLA-A2 (excluding HLA-A*0,203) HLA-A2	Recruiting Not yet recruiting	Triple Negative Breast Cancer
TRAIL^h^	TAA^l^	NCT05357027	2022.08	2024.08	ⅠⅡ	China	HLA-A2	Recruiting	Cervical Carcinoma
PRAME^i^	TAA	NCT05973487	2024.02	2026.12	Ⅰ	United States	HLA-A*02:01	Recruiting	Head and Neck Cancer Cervical Cancer Non-small Cell Carcinoma

^a^ Human leukocyte antigen.

^b^ HumanPapillomavirus.

^c^ Hepatitis B virus.

^d^ Epstein-Barr virus.

^e^ Cytomegalovirus.

^f^ Kirsten rats arcomaviral oncogene homolog.

^g^ Melanoma-associated antigen.

^h^ TNF-related apoptosis inducing ligand.

^I^ Preferentially expressed antigen melanoma.

^j^ Tumor-specific antigen.

^k^ Cancer germline antigen.

^l^ Tumor associated antigen.

Here, we discuss the structure and function of TCR-T cell therapies and suggest possible solutions by outlining the challenges of TCR-T for solid tumors and new strategies for innovative coupling of TCR-T with today’s popular therapeutic approaches. This will bring hope that subsequent TCR-T can be effective in defeating solid tumors.

## 2 The structure and function of TCR-T cells

The TCR molecule is a heterodimer made up of two transmembrane polypeptide chains joined by different disulfide linkages found on T cells’ surface. It enhances immunological responses and performs the specialized function of antigen recognition. The four peptide chains that make TCRs are α, β, γ, and δ. Most mature T cells have TCR molecules made up of α and β chains, but some also have TCR molecules made up of γ and δ chains. Less than 5% of all T cells in the circulation are γδ T cells (Pang et al., 2023; Zhang and Wang, 2019). TCR αβ cells recognize antigenic peptides presented on the surface of antigen-presenting cells (APCs) by class I or II MHC molecules in a selective manner. After being recognized, they grow and differentiate into effector cells, which produce cytokines or carry out cytotoxic actions. This process helps the body defend against pathogenic invasion and tumor growth by stimulating B cells or innate immune cells (Muro et al., 2019). On the other hand, TCR γδ cells are considered innate immune cells since they do not require sophisticated activation processes and are not restricted to the MHC (Hayday, 2019). During the immune response, γδ T cells may play a role in initiating, coordinating, and complementing αβ T cell functions (Park et al., 2022). T cells bearing αβ-type TCRs hold a pivotal role in adaptive immunity. Immunological surveillance by T cells stems from the process wherein peptide fragments of degraded intracellular proteins are transported into the endoplasmic reticulum, where they bind to self-recognized proteins. A recent study has unveiled the high-resolution cryo-electron microscopy structure of a human TCR-CD3 complex containing eight subunits, offering a comprehensive molecular insight into the complex (Dong et al., 2019). Consistent with earlier biochemical data, the TCR-CD3 complex consists of an antigen-recognition module of disulfide-bonded TCRα/β heterodimers and three CD3 dimers, including CD3γε and CD3δε heterodimers, and a CD3ζζ homodimer, with a stoichiometry of 1:1:1:1 (Call et al., 2004).

An immunoreceptor tyrosine (ITAM) activation motif and an extracellular immunoglobulin (Ig) superfamily structural domain are present in each CD3γ/δ/ε subunit. On the other hand, CD3ζ has three ITAMs and a brief extracellular structural domain (ECD). As a result, a complete TCR-CD3 complex with 10 ITAMs has 20 tyrosine phosphorylation sites, which allows for reactions to various antigenic stimuli (Courtney et al., 2018). To activate T cells (Signal 1) and produce co-stimulatory molecules (Signal 2), which work in concert to increase the activity of activated cells, the TCR-CD3 complex initiates a signaling cascade. This mechanism helps cytotoxic T lymphocytes recognize and destroy sick or cancerous cells (Banchereau and Steinman, 1998; Chen and Flies, 2013). A secondary activation signal for T cells necessitates the involvement of co-stimulatory receptors. For instance, CD28 triggers T cell activation, and upon binding to its ligands B7 (CD80 and CD86) on the surface of APCs (Chen and Flies, 2013), it enhances TCR-driven tyrosine phosphorylation. This activation process recruits phosphatidylinositol 3-kinase (PI3K) to collaborate with growth factor receptor-bound protein 2 (Grb2) (Rudd et al., 2009). In the absence of CD28 engagement, TCR activation often results in an anergic state, characterized by functional inactivation of T cells upon antigen encounter, albeit they persist for a period in a hyporesponsive state (Linsley and Ledbetter, 1993). The collective impact of these signals dictates T-cell expansion, memory formation, and functional persistence (Rath and Arber, 2020).

TCR signaling strength is usually correlated with peptide-MHC (pMHC) binding affinity (Sibener et al., 2018), and T cell signaling constraints dictate a consensus TCR-pMHC docking topology that is highly conserved, enabling canonical TCR-pMHC I docking to localize CD8/Lck to CD3 complexes optimally (Zareie et al., 2021). However, this process can be constrained by the reversal of TCR-pMHC polarity, a phenomenon intricately involved in immune responses (Gras et al., 2016). TCR binding affinity to pMHC ranges from 500 μM to 1 μM (Huppa and Davis, 2003). Mechanical force initiates dynamic mechano-chemical coupling, leading to sequential alterations in agonist pMHC conformation. In this process, the TCR establishes a capture bond with agonist pMHC while forming a slip bond with non-agonist pMHC ligands (Sibener et al., 2018; Wu et al., 2019). Different tumor tissues form with different stiffnesses, especially solid tumors, generating mechanical forces that may directly affect the TCR-pMHC capture bond dynamic structural model. Cancer-associated somatic mutations or subtle polymorphic changes in HLA class I inhibit TCR-pMHC capture bond formation and reduce T cell recognition of cancer cells (Sibener et al., 2018).

**FIGURE 1:**
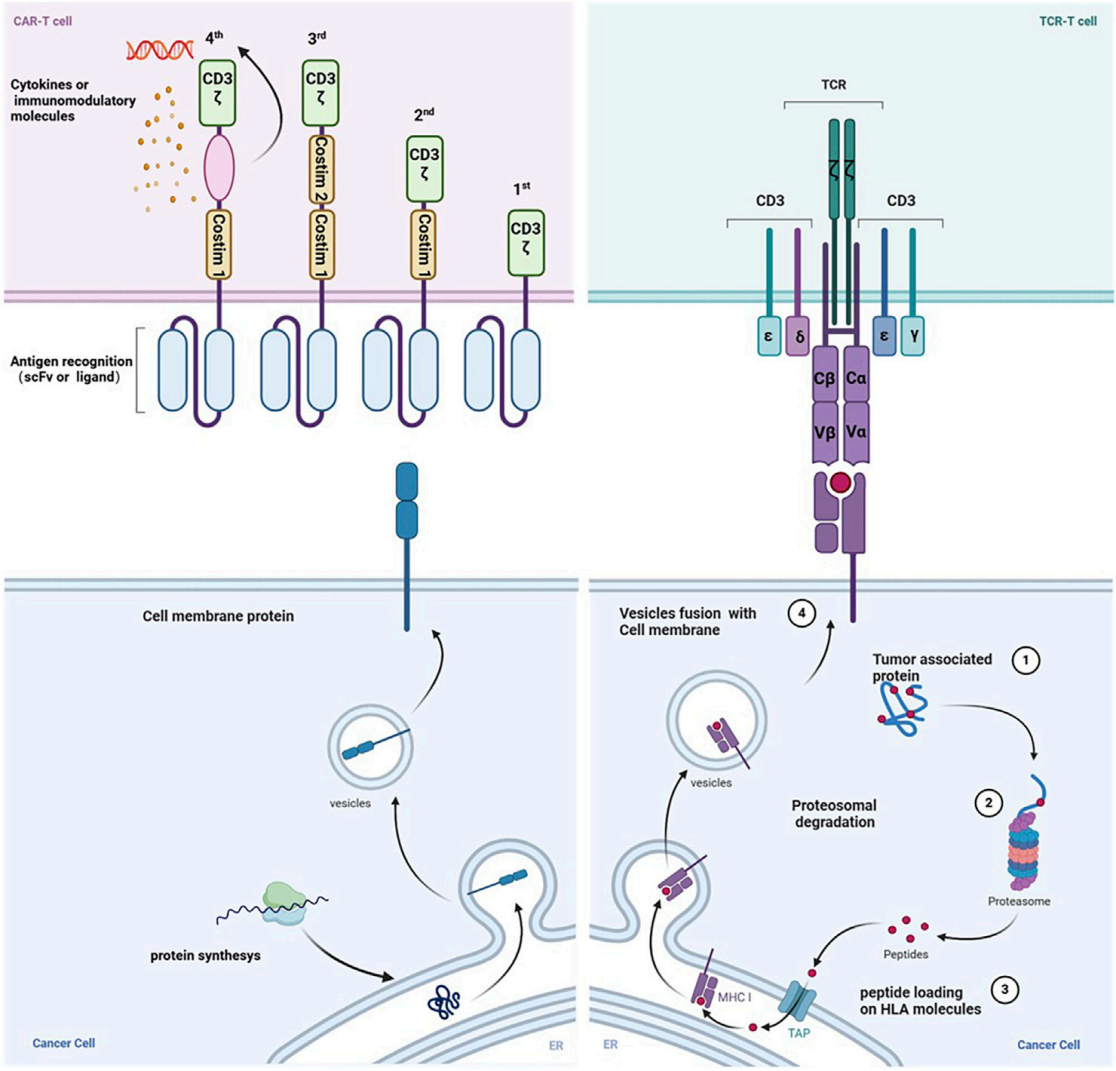
The mechanism of action differs between CAR-T cells and TCR-T cells. The generations of chimeric antigen receptors (CARs) and their structural distinctions are illustrated. CARs typically identify surface proteins using antibody-derived scFv recognition structural domains. Conversely, TCR T-cells operate on the principle of modifying T-cells’ natural receptors to improve cancer cell recognition. The structure of the endogenous or genetically engineered T-cell receptor (TCR)-CD3 complex can recognize peptides presented by HLA molecules originating from various cellular compartments. Created with BioRender.com.

TCR and CAR-engineered T cells are manipulated *ex vivo* using peripheral blood from either patients or healthy donors. Following expansion in culture to attain adequate cell numbers, these engineered T cells are reintroduced into patients to target and eliminate cancer cells (Figure 1). However, they exhibit distinct mechanisms for antigen recognition (Table 2). In contrast to CARs, TCRs display greater sensitivity in antigen recognition. The TCR-pMHC interaction process demonstrates remarkable specificity, heightened sensitivity, and rapid biochemical reaction kinetics. Under physiological conditions, tumor cells carry abundant self-antigens and hidden tumor antigens to achieve antigen escape. TCR-T accurately differentiates between abnormal and low concentrations of pMHC ligands and triggers an adaptive immune response in the presence of most autoantigenic interferences (Feinerman et al., 2008). If pMHC is in tandem with multiple TCR molecules, a more significant stimulus signal can be generated (Valitutti, 2012). TCR-T cells release fewer cytokines compared to CAR-T cells. While CAR T cells have demonstrated effectiveness as effector cells targeting the same malignancy, they exhibit transiently elevated cytokine levels, increasing the risk of cytokine storms. Conversely, TCR-T cells exhibit superior expansion capabilities under heightened antigen exposure to CAR-T cells. They also pose a reduced risk of cytokine storm, exhibit diminished expression of co-inhibitory molecules, better navigate the immunosuppressive microenvironment of solid tumors, and sustain T-cell activity to efficiently eradicate tumors (Wachsmann et al., 2022).

**TABLE 2:** Comparison of car-t and tcr-t.

	Factors	CAR-T	TCR-T
Common ground	Genetic modification	Difficult, expensive	Difficult, expensive
	Procedure of treatment	Reinfusion after *in vitro* modification	Reinfusion after *in vitro* modification
	Specificity	High	High
	Immune suppression	Overcoming efficiently	Overcoming efficiently
	Flexibility	Less	Less
Distinction	Structure	An engineered receptor composed of intracellular and extracellular domains	Native or minimally designed TCR
	Core advantage	Cell surface antigen (no MHC restriction)	Intracellular antigen (pMHC restriction)
	Number of ITAMs	Three	Ten
	Adverse effect	Cytokine release syndrome; neurotoxicity	Cytokine release syndrome; neurotoxicity
	Tumor applications	CAR-T therapy has taken a leading position in the field of hematologic malignancies	TCR-T therapy places greater emphasis on research and development within the field of solid tumors

## 3 Limitations of TCR-T therapy for solid tumor treatment

**FIGURE 2:**
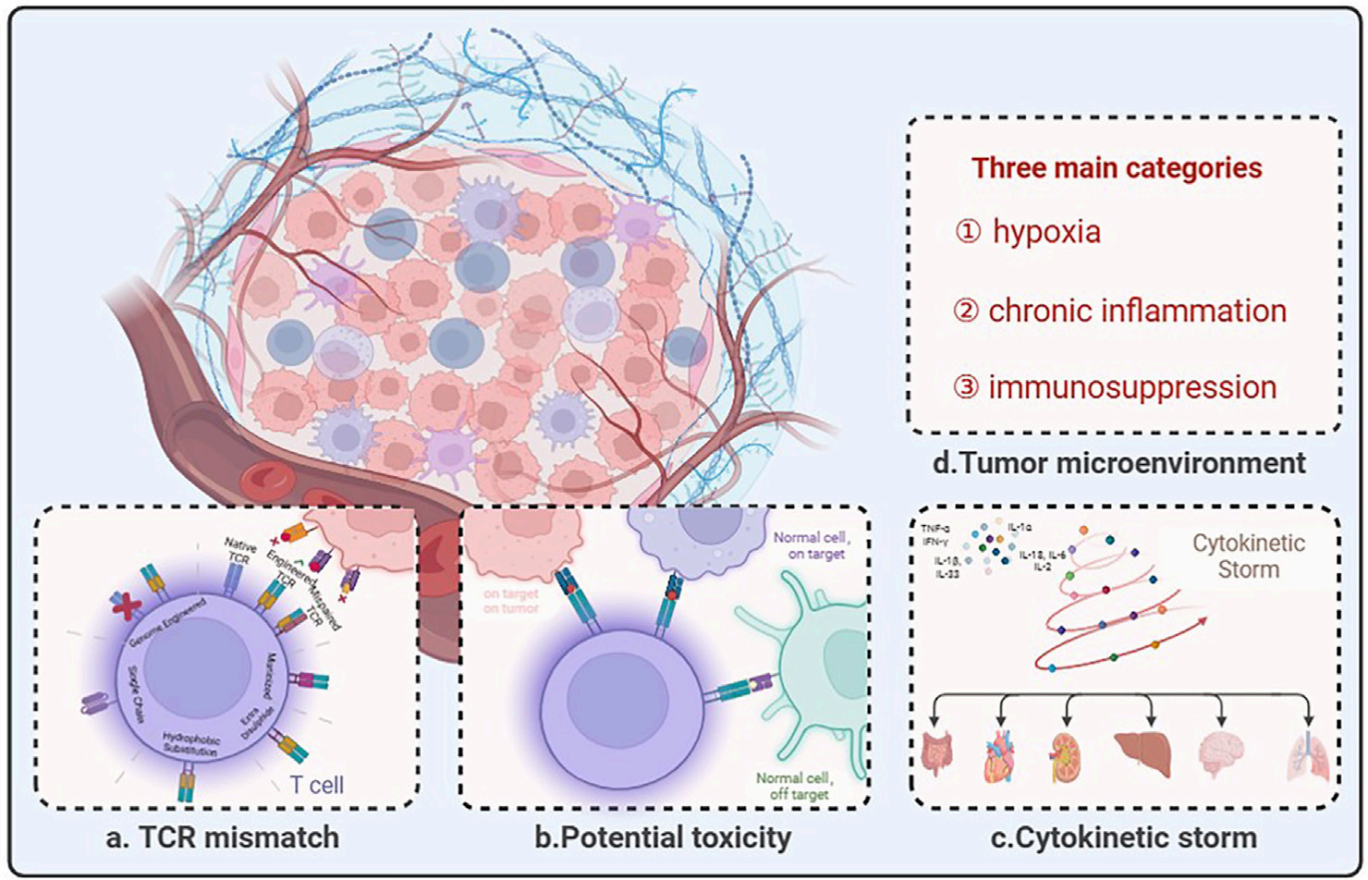
The predicament of TCR-T therapy in solid tumors lies in its limited efficacy due to several challenges. **(A)** Description of pairing errors between endogenous and engineered TCRs and potential strategies. **(B)** TCR-modified T cells are designed to redirect antigenic responses and maintain specificity, but at the same time TCR-engineered T cells have the potential to: “On target, on tumor”: appropriate antigen recognition leading to tumor eradication; “Normal cell, on target”: TCR-T recognizes low-level antigens on normal tissues; “Normal cell, off-target”: TCR-T recognizes relevant or irrelevant antigens on target or non-targeted tissues. **(C)** Cytokine storm is an immune dysregulation disorder encompassing several conditions characterized by systemic symptoms, systemic inflammation, and multiorgan dysfunction, which may lead to multiorgan failure if not properly treated. **(D)** The tumor microenvironment comprises three main categories: hypoxia, chronic inflammation, and immunosuppression. Created with BioRender.com.

The current TCR-T for solid tumors dilemma can be divided into four segments: TCR mismatch and multiple limitations of targets, potential toxicity, cytokinetic storm, and tumor microenvironment (Figure 2). Overcoming these challenges will be key to constructing TCR-T cells capable of recognizing reliable targets with sufficient affinity and function to eliminate existing tumors and prevent recurrence.

### 3.1 TCR mismatch

The mismatch between exogenous and endogenous TCR chains has been a non-negligible problem for engineered TCR-T cell therapy. Exogenous TCR chains can have a competitive relationship with endogenous TCR chains, and exogenous TCR chains introduced after screening have a high affinity to dominate (Heemskerk et al., 2007). The introduction of exogenous TCRs into T cells has several effects, including the formation of mixed TCR dimers. However, the properties of different mixed TCR dimers are unpredictable and may affect the subsequent biological activity of T cells. These dimers might prevent transferring TCR-T cell therapy to clinical applications. It has been shown that TCR transfer leads to the generation of hybrid TCR dimers of unknown specificity, which may cause these T cells to cannibalize each other and exhibit novel deleterious reactivity, such as graft-versus-host response (GVHD) (van Loenen et al., 2010). Mixed TCR dimers may compete with engineered TCR heterodimers for binding of restricted CD3 components, resulting in off-target presentation of new antigenic peptides to the surface and triggering off-target effects. In the absence of the ability to eliminate mixed dimers, cysteines were used to design TCR constant regions of the exogenous chain, increasing the total surface expression of the introduced TCR chain, reducing the extent of mismatch, and decreasing the risk of developing graft-versus-host disease (Kuball et al., 2007). Enhanced TCR chain expression using hybridized human TCR chains containing mouse constant structural domains does not bind to endogenous TCR chains containing human constant structural domains (Chen et al., 2017). However, immunogenicity can limit T-cell viability to some extent (Cohen et al., 2006). In contrast to phage display methods, since the binding affinity of TCRs or mutants expressed on yeast cells can be directly assessed, yeast surface display technology screens for high-affinity TCRs and reduces the chance of TCR mismatch (Smith et al., 2015; Shafer et al., 2022). By introducing an additional stable disulfide bond between residue 48 of the TCR constant region α and residue 57 of the TCR constant region β through cysteine substitution, the inter stream binding affinity of the engineered TCR α/β chains is enhanced while simultaneously reducing their binding affinity to the endogenous TCR α/β chains (Krshnan et al., 2016). The stability of the engineered TCR α chain can be enhanced through the selection of hydrophobic substitutions in the transmembrane region (Haga-Friedman et al., 2012). Single-chain TCR (scTCR) entails the incorporation of TCR antigen recognition and signaling domains into a singular chain, thereby minimizing mismatches through spatial site-blocking (Knies et al., 2016).

### 3.2 Multiple limitations of targets

For a considerable time, tumor-specific antigens, or TSAs, have been considered ideal targets for cellular immunotherapy. Nonetheless, the existing repertory of TSAs against solid tumors is still restricted, which reduces the effectiveness of traditional CAR-T cell therapy. On the other hand, the advantage of TCR-T treatment is that it targets both intracellular and cell surface antigens, greatly expanding the pool of potential targets. Notwithstanding this promise, very few peptide antigen targets have been shown to be both safe and efficacious for TCR-T immunotherapy. Research to detect immunogenic neoantigens in tumor cells may provide a significant discovery. Tumor cell-produced neoantigens need to be unique to tumor cells, not expressed in normal cells, and have high expression levels so that MHC molecules can recognize them (Wang and Cao, 2020). Not all people produce neoantigens in the same way, and even individuals with the same type of solid tumor have diverse tumor cells that express different antigens. Individual differences in neoantigens need the creation of customized TCR-T cell therapy regimens, which are closely related to the financial and schedule aspects of TCR-T cell therapy (Li et al., 2023a). Clear studies on the stability of neoantigens are lacking, posing challenges to mitigating the risk of neoantigen loss. Downregulation or loss of MHC class I molecules on tumor cells diminishes the sensitivity of TCR-T therapy and serves as a prominent pathway for tumor evasion (Dutta et al., 2006; Singh and Banerjee, 2020; Xu et al., 2022). The targets of TCR-T cell therapies are further constrained by MHC type. HLA genes encoded MHC molecules exhibit extensive diversity within the population, with over 20,000 HLA I human alleles identified to date (Gonzalez-Galarza et al., 2020). At present, the research field of TCR-T therapy is constantly exploring new targets to improve the therapeutic effect and expand the therapeutic scope. The research and development of novel targets mainly include tumor-specific neoantigens (Xie et al., 2023), virus-associated antigens and cancer-testis antigens (Gordeeva, 2018) (Table 1).

### 3.3 Potential toxicity

Regarding the dynamics of therapy, TCR-T therapy is more sensitive than CAR-T therapy, making it more vulnerable to non-tumor-targeted toxicity (on-target off-tumor) and cross-reactivity (off-target off-tumor). The former is related to the expression of target antigen in normal tissues, especially in the case of TAA. The latter refers to the fact that the TCR recognizes different antigens on normal cells, especially when the affinity of the TCR sequence is enhanced. This vulnerability may cause damage to normal human tissues that have comparable antigenic epitopes. An illustrative instance involves the treatment of myeloma and melanoma patients with engineered T cells targeting the affinity-enhancing TCR of MAGE-A3, resulting in unforeseen occurrences of cardiogenic shock and mortality. In these cases of myocardial injury, histopathological analysis revealed the infiltration of T cells but no expression of MAGE-A3 was detected in cardiac autopsy tissue. Because TCR-T cells recognize an unrelated peptide from the rhabdomyosarcoma-specific protein titin, it is thought to be due to cross-reactivity (Linette et al., 2013). In a clinical investigation, highly reactive transferred TCR-T cells transported to melanoma tumors in patients and destroyed melanoma in their bodies, but patients showed damage to normal melanocytes in the skin, eyes, and ears (Johnson et al., 2009). Another study delineates instances of severe acute colitis induced in patients with metastatic colorectal cancer who were treated with TCR-T cells targeting carcinoembryonic antigen (CEA). This outcome arose from the expression of the target antigen on normal intestinal tissue as well (Parkhurst et al., 2011). The concept of TCR affinity embodies a paradox. It is widely acknowledged that robust affinity is imperative for sustaining T-cell expansion and facilitating the regression of human cancers. Excessive affinity, however, can cause cross-reactivity with self-antigens by targeting healthy cells across the body that carry homologous antigens and prematurely exhausting T cells.

### 3.4 Cytokinetic storm

Cytokine storm represents a spectrum of clinical disorders marked by significant health risks arising from the excessive production of inflammatory factors by cell therapy. This phenomenon encompasses manifestations such as fever, tachycardia, hypotension, rash, and respiratory distress, emerging as the predominant adverse reaction to T-cell immunotherapy. Key cytokines implicated in a cytokine storm, including Interferon-gamma, IL-1, IL-6, TNF, and IL-18, exhibit consistently elevated levels and are believed to orchestrate the central pathological mechanisms underlying cytokine release syndrome (CRS). Since TCR-T can recognize peptide epitopes derived from proteins in any subcellular (e.g., membrane, cytoplasmic, and nuclear) compartment (Chandran and Klebanoff, 2019), has a broader range of antigen selection, and the TCR chain has intrinsic signaling and regulates T cells, resulting in a lower rate of CRS as compared to CAR-T cells, T cell receptor (TCR)-T cells are also considered promising immunotherapy. Low incidence is not the same as complete avoidance. Mikiya Ishihara et al. utilized retroviral vectors to engineer precise silencing of endogenous TCRs and induce the forced expression of the affinity-enhancing NY-ESO-1-specific TCR in T cells. The system expresses small interfering RNAs (siRNAs) that are specific to endogenous TCR genes to enhance the expression of transduced tumor-specific TCR while minimizing potential TCR mispairing. In patients with tumors that highly express NY - ESO - 1, the infusion of TCR - T cells led to a significant tumor response as well as early-onset CRS (Ishihara et al., 2022). Similarly, a patient with fallopian tube cancer, unresponsive to adjuvant chemotherapy, participated in a clinical trial of MAGE-A4-targeted T-cell receptor T-cell therapy. Following T-cell infusion, the patient experienced CRS and pseudogouty arthritis accompanied by immune effector cell-associated neurotoxicity syndrome (ICANS) within 7 days. Notably, the drug tocilizumab was ultimately utilized to eliminate CRS and ICANS successfully (Kim et al., 2021). Hence, to enhance the safety and efficacy of TCR-T cell immunotherapy, it is imperative to prudently evaluate the significance of cytokine storm risk for patient prognosis.

### 3.5 Tumor microenvironment

The first obstacle genetically modified T cells must overcome while fighting solid tumors is to infiltrate the tumor’s hostile environment successfully. Solid tumors reinforce their conquered area by creating complex and formidable ecosystems. Cancer cells are remarkably adaptive as tumors progress, repurposing various non-tumor cell types to create an environment that supports their growth and survival (Heindl et al., 2015; Wang S. et al., 2020). The tumor microenvironment comprises three main categories: hypoxia, chronic inflammation, and immunosuppression (Policastro et al., 2013). Within the relatively constrained confines of solid tumors, hypoxia emerges as a pervasive condition, significantly impacting the rapid proliferation and metabolic activity of T cells. While CD8^+^ T cells serve as the cornerstone of tumor elimination, their vigor wanes, and exhaustion sets in when confronted with hypoxic conditions. Recent discoveries have unveiled a paradox: as this fundamental component becomes terminally exhausted, it transforms, upregulating CD39 expression to foster an immunosuppressive milieu that undermines the robust anti-tumor capabilities of other T cells (Vignali et al., 2023; Canale et al., 2018). Tumor-associated macrophages (TAMs) constitute pivotal constituents of the tumor microenvironment. Studies in hepatocellular carcinoma therapeutics have unveiled a robust correlation between downregulation of xanthine oxidoreductase (XOR) expression and specific tumor microenvironment traits, particularly hypoxia (Ullah et al., 2024b; Ullah et al., 2024c). Deletion or dysfunction of XOR in monocyte-derived macrophages fosters CD8+ T cell depletion by inducing the upregulation of immunosuppressive metabolites (Lu et al., 2023). Most tumor stromal cells within the TME exhibit numerous immunosuppressive signaling proteins, including programmed death ligand-1 (PD-L1). The interaction between PD-L1 and programmed cell death protein-1 (PD-1) (Noman et al., 2014) expressed in T cells can result in immune cell dysfunction and apoptosis (Dermani et al., 2019). Hypoxia induces elevated potassium levels and an acidic milieu, disrupting potassium homeostasis in tumor-infiltrating lymphocytes (TILs) within necrotic tumor microenvironment regions (Conforti, 2017). This severely suppresses T-cell effector function, impacting cytokine secretion capacity and activity (Eil et al., 2016).

## 4 Innovative association

**FIGURE 3:**
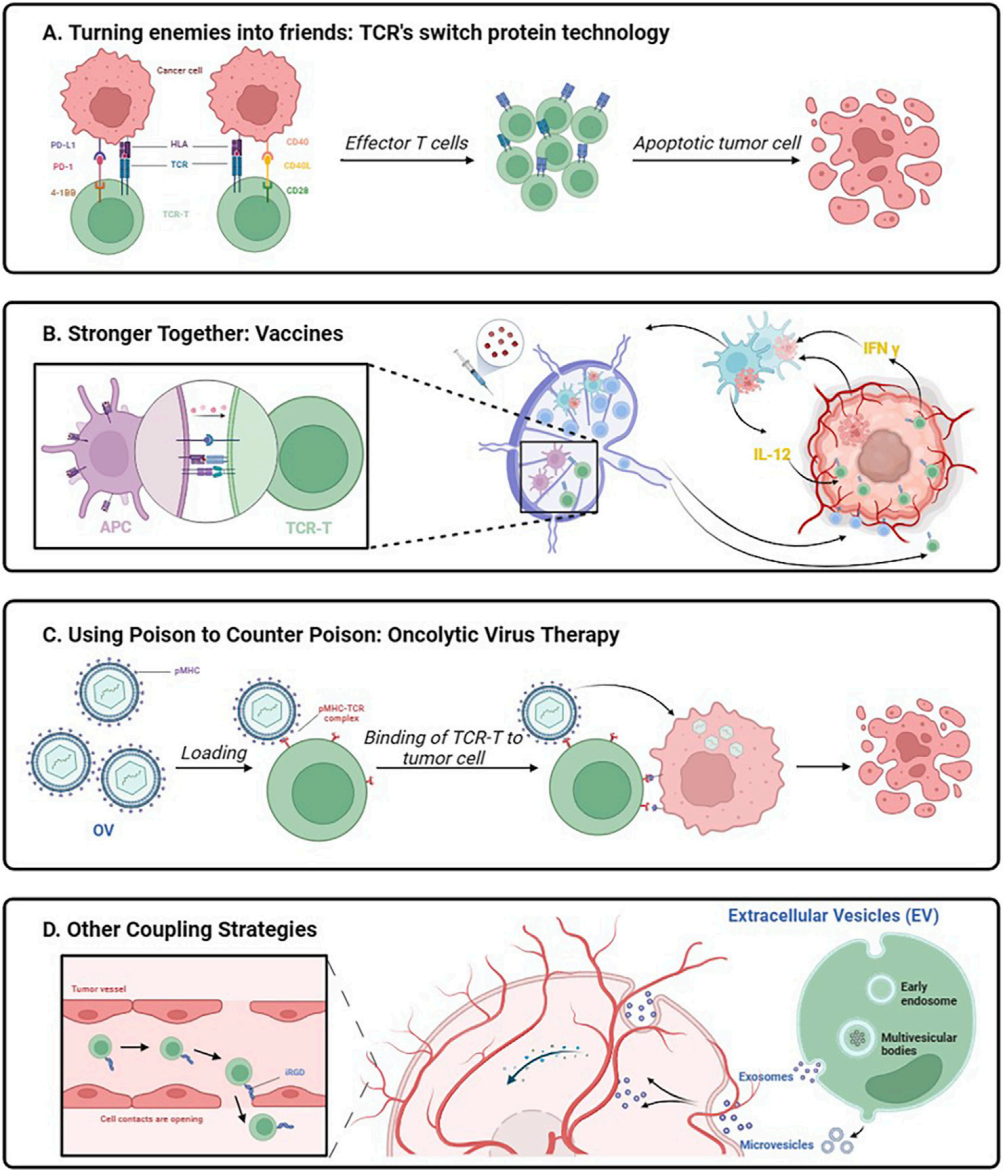
Schematic diagram of innovative association working mechanism. Created with BioRender.com.

TCR-T therapy needs to improve T cell expansion and persistence *in vivo* to avoid rapid loss of effector function. Because TCR-T alone may not be sufficient to generate an effective antitumor response, innovative combination strategies may improve response rates in patients with high tumor loads and reduce the need for large numbers of TCR-T cells as a potential approach to improving clinical outcomes (Figure 3).

### 4.1 Turning enemies into friends: TCR’s switch protein technology

The PD-1/PD-L1 inhibitory axis represents an immune checkpoint mechanism evolved by the body to regulate the magnitude and duration of immune responses, thereby preventing excessive inflammatory reactions and autoimmune diseases that may harm healthy tissues (Dai et al., 2014; Dyble et al., 2015). In the endeavor to corral tumor cells, the PD-1/PD-L1 inhibitory axis is a vulnerability. Tumor cells accomplish immune evasion by elevating PD-L1 ligand expression and inhibiting T cell function through PD-1 engagement. PD-L1, functioning as a natural ligand for PD-1 (Ji et al., 2019), assumes the role of a guardian for tumor cells, endowed with the capability to convey anti-apoptotic signals and foster tumor proliferation (Dong et al., 2018).

When this route is unchecked, T cells’ ability to fight cancer is compromised. To prevent the PD-1/PD-L1 pathway, scientists have developed a variety of PD-1/PD-L1 immune checkpoint inhibitors. These inhibitors have been successfully used to treat a variety of malignancies, including classical Hodgkin’s lymphoma, melanoma, non-small cell lung cancer, and squamous cell carcinoma of the head and neck. Moon EK et al. reported that the combination of TCR-T cell therapy with PD-1 antibody reduced hypofunctionality in tumor-infiltrating lymphocytes (TILs) and augmented the efficacy of overt metastatic T cells under conditions of chronic antigenic stimulation (Moon et al., 2016). At present, cellular immunotherapies engineered to produce autocrine PD-1 antibodies or to suppress PD-1 expression are available for treating solid tumors (Hamilton et al., 2023; Wu et al., 2023; Zhang et al., 2022). PD-1/PD-L1 immune checkpoint inhibitors provide advantages but also significant hazards. Studies reveal significant effects on the heart, resulting in serious illnesses such as myocarditis, stress cardiomyopathy, pericardial disease, and an increased risk of complications associated to the heart’s immune system (Varricchi et al., 2017; Costa et al., 2017; Khunger et al., 2020).

If playing hardball proves ineffective, why not transform adversaries into allies? The EtoE platform introduces an inherent 2-in-1 combination therapy (Schendel, 2023). The pivotal element of this therapy lies in the utilization of the PD1-41BB co-stimulatory switch protein (CPS) incorporated into TCR-T cells, enabling these cells to concurrently express recombinant TCR and PD1-41BB switch receptors (Sailer et al., 2022). The ingenuity of the concept resides in substituting the inhibitory signaling structural domain of PD-1 with the activating signaling structural domain of 4-1BB while retaining the external structural domain of PD-1 responsible for binding to PD-L1 on tumor cells. The intracellular signaling domain of the 4-1BB protein offers a comprehensively characterized pathway for co-stimulation, augmenting T-cell responses, and activating various immune cells (Vinay and Kwon, 2014). Therefore, by not impeding PD-1 binding to PD-L1, PD1-41BB-modified TCR-T cells exhibit enhanced tumor cell killing even in the presence of PD-L1-overexpressing tumor cells. This scenario converts potent inhibitory signals into secondary activating signals, prompting T-cell proliferation, cytokine expression, and bolstering T-cell effector function (Schlenker et al., 2017). The PD1-41BB CPS efficiently regulates the self-defense mechanism of tumor cells.

Another co-stimulatory switch protein targets the CD40L/CD40 pathway and the CD28 co-stimulatory signal. The CD40 receptor and its ligand, CD40L, constitute one of the most crucial molecular pairs of stimulatory immune checkpoints. CD40 binds to its ligand, CD40L, which is transiently expressed in T cells and other non-immune cells under inflammatory conditions (Elgueta et al., 2009). Initially characterized on B cells (Sacco et al., 2023), CD40 is expressed on professional antigen-presenting cells as well as non-immune cells and tumors (van Kooten and Banchereau, 1997). The CD40L-CD28 CPS is composed of an extracellular CD40L structural domain combined with an intracellular signaling structural domain of the CD28 co-stimulatory receptor (Olguín-Contreras et al., 2021). The principles related to the chimeric design of this CPS are not yet clear, and it is still unknown whether it can successfully achieve biological effects (Oda et al., 2017). CD40L-CD28 co-stimulatory switching protein-modified TCR-T cells provide a variety of new attributes that can contribute to the enhancement of cellular immune responses. As multiple cells in the tumor microenvironment express CD40, CD40L-CD28 engineers enhanced T-cell infiltration, and CD40L activates CD40-expressing immune cells to amplify anti-tumor responses. The presence of the CD28 signaling structural domain in the CSP provides further stimulation of TCR-T cells, improving cytokine secretion and antigen-specific cytotoxicity. This intrinsic combination enhances the function of TCR-T cells, alters the strong inhibitory nature of the tumor microenvironment against T cells, and mobilizes immune cells in the tumor microenvironment to participate in the battle against solid tumors (Schendel, 2023).

### 4.2 Stronger together: vaccines

Although TCR-T cell therapy has great promise in treating solid tumors, it still confronts several obstacles and frequently produces unsatisfactory clinical results. TCR-T therapy faces significant challenges in efficiently treating solid tumors due to factors such low T-cell infiltration capacity, hostile tumor milieu for T-cell activation, and limited *in vivo* durability of infused TCR-T cells. Permissive T cells can be genetically engineered to express different pro-inflammatory molecules, such as IL-12 (Liu et al., 2019; Pegram et al., 2012), IL-15 (Nguyen et al., 2023), IL-18 (Avanzi et al., 2018), CD40L (Kuhn et al., 2019), or DC growth factor FLT3 (Zhu et al., 2024; Lai et al., 2020). These modified T cells have the ability to self-supply pro-inflammatory chemicals, promote epitope dissemination, and enhance T cells’ anti-tumor activity *in vivo*. These tactics are supported by the fact that transformed T cells are viable and abundant *in vivo* and that T-cell death reduces the release of pro-inflammatory chemicals. However, because the *in vitro* production method takes time, there is a chance that genetically altered T cells will not be easily accessible for subsequent infusion. In contrast, the simplicity of tumor vaccine preparation and the ability to control the infusion timing outweigh these strategies. Consequently, the combination of vaccines with TCR-T cell therapy holds promise in overcoming the lackluster outcomes associated with TCR-T cell therapy in the treatment of solid tumors.

While co-stimulatory signaling plays a crucial role in T cell activation, the downregulation of co-stimulatory ligand expression within the tumor microenvironment presents a challenge by restricting T cell activation. Attempts to enhance TCR-T efficacy through combined vaccination in early TCR-T trials did not yield the anticipated results in providing both TCR stimulation and co-stimulation *in vivo* (Nowicki et al., 2019; Kageyama et al., 2015). Significantly, the integration of vaccines with overt cellular therapies effectively triggers the activation of the endogenous immune system, facilitating the generation of host T cells targeting additional tumor antigens, thus preventing antigen-negative tumor escapes (Author anonymous, 2023; Mateus-Tique and Brown, 2023; Corbière et al., 2011). The phenomenon, termed “Antigen Spreading” (AS), has the potential to enhance the efficacy of TCR-T cell therapy for solid tumors. Recent studies conducted by Elicio Therapeutics have demonstrated that the combination of amphiphilic (AMP) vaccination, which selectively targets homologous peptides and adjuvants to lymph nodes, induces dendritic cell activation. This activation not only enhances T-cell activation through endogenous co-stimulation and cytokine-supported delivery of TCR-stimulants but also has the potential to trigger the collaboration of newly generated tumor-specific T cells with TCR-T cells, thus collectively combating tumors (Drakes et al., 2024).

In order to facilitate the targeted growth of T cells *in vivo*, customized vaccinations can also be made to target certain antigens of interest called neoantigens (Hellmann and Snyder, 2017). Personalized vaccinations can undoubtedly enhance antigen-specific T cells *in vivo*, as evidenced by research done on human HLA transgenic mice and cancer vaccine trial subjects (Sahin et al., 2017; Kunert et al., 2016). However, the combination of TCR-T cell treatments and cancer vaccines has only been studied in a small number of clinical trials, thus more study is needed to determine their therapeutic efficacy and develop a thorough and well-defined rationale.

### 4.3 Using poison to counter poison: oncolytic virus therapy

Immunotherapy against the oncolytic virus (OV) is a new and exciting treatment approach. Oncolytic viruses, whether natural or recombinant, have the ability to preferentially infect tumor cells, multiply within the tumor cells, and lyse tumor cells directly while sparing healthy cells. T-cell treatment with an oncolytic virus combination shows a synergistic impact. Targeting by lyssaviruses can compensate for the deficiency in T-cell trafficking (Kaufman et al., 2015), converting the “cold” tumor microenvironment into a “hot” one (Bommareddy et al., 2018). Moreover, it inhibits tumor immune evasion mechanisms and angiogenesis (Streby et al., 2017), achieving multifaceted tumor eradication. In addition to eliciting classical apoptosis and pyroptosis by targeting tumor antigens, it can induce autosis, a previously undocumented form of cancer cell demise. A study report delineates the synergy between an oncolytic virus and adoptive cellular therapies (CAR-T, TCR-T), effectively directing its attack toward drug-resistant cancer cells, ushering in new optimism for combating treatment-resistant cancers (Zheng et al., 2022).

### 4.4 Other coupling strategies

Tumor-homing cell-penetrating peptides (CPPs) constitute small amino acid sequences, typically short peptides (comprising less than 30 residues) (Ye et al., 2016), capable of traversing cell membranes. These peptides serve as effective vehicles for intracellular delivery, both *in vitro* and *in vivo*, facilitating the transportation of diverse biologically active cargoes, ranging from nucleic acids to large proteins and other compounds, with molecular weights extending up to 120 kDa. CPP enhances the penetration of small molecule drugs and nanoparticles into tumor tissues (Silva et al., 2019). Naiqing Ding et al. achieved enhanced infiltration of tumor-specific T cells through the rapid modification of T cells with the tumor-penetrating peptide iRGD using a lipid insertion approach (Ding et al., 2019). This method circumvents the need for intricate gene editing procedures. A novel bifunctional drug, iRGD-anti-CD3, tightly bridges internalizing RGD (Arg-Gly-Asp) peptide (iRGD) and T cells by binding to CD3. iRGD-anti-CD3-modified T cells not only alleviate the challenging infiltration of T cells in the tumor microenvironment but also induce T-cell activation and cytotoxicity against cancer cells (Zhou et al., 2021). Using CPP in conjunction with TCR-T cell treatment is a novel approach that has the potential to eventually improve the therapeutic efficacy of off-the-shelf cell therapy.

A common and serious side effect of cell treatment is called CRS, which is caused by immune cells releasing cytokines out of control. Cytokine storm is characterized by its intensity and presents serious dangers, including organ failure and death. Researchers have started looking into the use of genetically altered exosomes made from altered cells in cancer treatment as a way to lessen this difficulty. Exosomes provide a significant benefit over using genetically changed post-modified cells directly because of their nanoscale size and innate ability to cross biological barriers, especially when it comes to solid tumor therapy. Exosomes have been shown in numerous studies to be able to be tailored for target specificity, which allows them to act as nanocarriers for anti-tumor medicines and antigen-specific anti-tumor immune responses (Roma-Rodrigues et al., 2014; Cheng and Hill, 2022). Therefore, exosome-based treatments show great therapeutic promise for cancer patients. Several examples of these therapies have been reported in preclinical models or clinical trials, including dendritic cell exosomes (DC-Exos) (Li et al., 2023b) and natural killer exosomes (NK-Exos) (Sun et al., 2020; Sun et al., 2019). Exosomes have advantages over cell-based therapies, including higher yield, increased safety, easier storage, and lower costs (Nag et al., 2023; Xu et al., 2020; Wang et al., 2016). Vδ2-T cells are known for their potential in anti-tumor and anti-infective capabilities, and they play a crucial role in recognizing and attacking both tumor and infected cells. Additionally, they are essential in controlling inflammatory pathways and immunological responses. A method of treating EBV-associated tumors with Vδ2-T-Exos, which possesses the cytotoxic and immunostimulatory characteristics of Vδ2-T cells, was suggested by Xiwei Wang et al. This strategy effectively controls EBV-associated tumors by combining the benefits of merging NK-Exos and DC-Exos (Wang X. et al., 2020). Exosomes produced from TCR-T cell culture can be used as a unique and potentially very effective cancer therapy approach. By acting as direct attackers and taking the place of TCR-T cells *in vivo*, they improve the safety and controllability of treatment.

## 5 Future perspective

TCR-T therapy holds significant promise for the treatment of solid tumors, yet various challenges must be addressed to fully realize its potential. There are related strategies to reduce TCR mismatch by interfering with endogenous TCRs. Tumor cells genetically engineered to express antigens from genetically modified T cells exhibit enhanced cytotoxic activity by specifically silencing endogenous TCRs and introducing tumor-specific TCR vectors (Ochi et al., 2011; Okamoto et al., 2009). Knockdown of its own TCR by gene editing (Georgiadis et al., 2018; Eyquem et al., 2017) or the use of the TCR γδ structural domain in TCRαβ to improve TCR pairwise binding (Hiasa et al., 2009; Parlar et al., 2019; Ishihara et al., 2023; Wang et al., 2023) to increase the level of functional exogenous TCRs on the surface of the T cells, which also avoids the risk of generating attacking self-antigens. The high cost of development and production of personalized TCR-T drugs has led to high market prices. How to reduce costs and achieve large-scale production will be the key to future commercialization. In a stride toward advancing scientific research, TCR-T cell therapies are transitioning towards the allogeneic generalization of this concept, departing from the customized design of individual antigens. The challenge of insufficient targets for TCR-T cell therapy has been addressed by a ground-breaking study that suggests using membrane-fused nanoparticles (NPs) as targets for specific recognition by TCR-T cells, regardless of the original HLA type, thereby modifying peptide-HLA (pHLA) onto the surface of tumor cells and allowing for the selective identification and elimination of tumor cells (Xu et al., 2023). Lower affinity T cells that have been developed might be a more secure option, but they frequently do not have the required antitumor effect. A potential solution to this conundrum is to introduce a suicide gene into TCR-T cells. This strategy prevents nonspecific damage to other tissues by maintaining effective tumor killing while inducing death in T cells at certain nodes. Another solution is to screen for TSA that is more tumor-specific than TAA, or to reduce the risk of toxicity and improve anti-tumor efficacy through affinity optimization and sequence modification. To counteract the diverse adverse conditions within the tumor microenvironment, engineered cytokines resistant to hypoxic and acidic environments commonly found in tumors are directed toward weakening the physical barriers of the extracellular matrix within the TME. This strategy aims to enhance T-cell infiltration capacity and augment the efficacy of adoptive T-cell therapies (Gaggero et al., 2022). Targeting TCR-T cells with immunosuppressive factors or enhancing the signaling function of intracellular signaling structural domains can reduce T cell depletion to counteract the immunosuppressive effects of tumors and become effective potential strategies. TCR-T therapy has the potential to become a transformative treatment for solid tumors.

## 6 Conclusion

Genetically engineered T cells represent a groundbreaking therapy for refractory tumors, capable of selectively eliminating cancer cells while sparing normal ones. TCR-T cell therapy and CAR-T cell therapy stand out among the various over-the-counter T cell therapies. However, CAR-T therapy often falls short in treating solid tumors, highlighting the superior potential of TCR-T cells. TCR-T cells offer unmatched advantages, as they can recognize epitopes from both surface and intracellular proteins, enabling the detection of a broader range of targets, including tumor-associated antigens (TAAs), cancer germline antigens, viral oncoproteins, and tumor neoantigens, compared to CAR-T cells. Furthermore, TCR-T cells require a broader range of affinities to activate T cells effectively compared to CAR-T cells. Thus, TCR-T cell therapy holds promising prospects for solid tumor treatment, with numerous studies progressing to the clinical stage.

Despite its potential, TCR-T cell therapy faces significant obstacles, including TCR mismatch, potential toxicity, cytokine storms, the tumor microenvironment, and target limitations. Various novel technologies and strategies are being applied to enhance the efficacy and safety of TCR-T therapy. These efforts primarily focus on reducing TCR mispairing to enhance TCR expression and function, augmenting the persistence and anti-tumor activity of engineered T cells, improving the homing and infiltration of engineered T cells into solid tumors, overcoming the immunosuppressive tumor microenvironment, and targeting neoantigens to enhance tumor-specific killing.

The innovative combination approach has sparked a research frenzy, with TCR-T cell therapy and other immunotherapies striving to identify the optimal “golden pair” through rational combinations, aiming for synergistic anti-tumor efficacy. However, developing innovative combinations poses significant challenges, with high barriers in the research and development process. While early studies provide valuable insights, a long road is still ahead before mature product development can be achieved. Additionally, TCR-T therapy requires further refinement and optimization of its production process to emerge as a potent tool in the fight against cancer and substantial tumors, ultimately revolutionizing cancer immunotherapy.
